# Berg Balance Scale Scoring System for Balance Evaluation by Leveraging Attention-Based Deep Learning with Wearable IMU Sensors

**DOI:** 10.3390/bioengineering12040395

**Published:** 2025-04-07

**Authors:** Zhangli Lu, Huiying Zhou, Honghao Lyu, Haiteng Wu, Shaohua Tian, Geng Yang

**Affiliations:** 1 State Key Laboratory of Fluid Power and Mechatronic Systems, School of Mechanical Engineering, Zhejiang University, Hangzhou 310027, China; 2 Dongfang Electric (Hangzhou) Innovation Institute Co., Ltd., Hangzhou 310000, China; 3 Zhejiang Engineering Research Center of Robotics in Electric Equipment Manufacturing and Intelligent Operation-Maintenance, Hangzhou 310000, China; 4 Zhejiang Key Laboratory of Intelligent Operation and Maintenance Robot, Hangzhou Shenhao Technology, Hangzhou 310000, China

**Keywords:** inertial measurement unit (IMU), Berg Balance Scale (BBS), gait analysis, deep learning, balance assessment

## Abstract

Balance assessment is crucial for health monitoring and rehabilitation evaluation of neurological diseases like Parkinson’s disease (PD) and stroke. The Berg Balance Scale (BBS) is a widely used clinical tool for balance evaluation. However, its dependence on trained therapists for subjective, time-consuming assessments limits its scalability. Current researchers have proposed several automated assessment systems. However, they suffer from difficulty in use in clinical settings and the need for feature engineering. The rapid advancement of wearable inertial measurement units (IMUs) provides an objective tool for motion analysis that is suitable for use in clinical environments. Thus, to address the limitations of manual scoring and complexities of capturing gait features, we proposed an automated BBS assessment system using an attention-based deep learning algorithm with IMU data, integrating convolutional neural networks (CNNs) for spatial feature extraction, bidirectional long short-term memory (Bi-LSTM) networks for temporal modeling, and attention mechanisms to emphasize informative features. Validated with 20 healthy subjects (young and elderly) and 20 patients (PD and stroke), the system achieved a mean absolute error (MAE) of 1.1627 and root mean squared error (RMSE) of 1.5333. Requiring only 5 min of walking data, this approach provided an efficient, objective solution for balance assessment to assist healthcare physicians as well as patients in their own health monitoring. The key limitations included: a limited generalizability to severely impaired patients who were unable to walk independently, and the inability to predict the score of individual tasks.

## 1. Introduction

Balance is a critical indicator of overall health, reflecting not only an individual’s physical stability but also offering insights into underlying medical conditions [1]. Adequate balance is essential for daily functioning and can serve as a marker for general well-being [2]. Moreover, variations in balance performance are closely linked to the progression or recovery of neurological conditions such as Parkinson’s disease (PD) and stroke, making balance assessment a valuable tool in clinical diagnostics and rehabilitation monitoring [3].

The Berg Balance Scale (BBS) is one of the most widely used clinical tools for evaluating balance [4]. It comprises a series of functional tasks that quantify a person’s balance ability and fall risk. However, traditional BBS assessments require a trained physical therapist (PT) to administer and interpret the test, making the process time-consuming and scoring subjective [5]. This reliance on expert evaluation limits the scalability and efficiency of BBS, particularly in settings that demand frequent monitoring or remote assessment.

Many researchers have developed systems for assessing BBS scores. Bacciu et al. [6] introduced a system that leverages the Wii Balance Board in conjunction with a neural network to predict BBS scores for participants. Johnson et al. [7] used Kinect 2 to get the image data of volunteers while doing certain tasks for BBS scores estimation and obtained good results. However, due to their susceptibility to environmental influences, they are not suitable for application in clinical settings.

The rapid advancement of wearable sensor technology provides an objective tool for automated balance evaluation that is suitable for use in clinical environments [8]. Inertial measurement units (IMUs) have emerged as a popular choice due to their ability to capture detailed, continuous motion data during everyday activities. These wearable devices enable objective, real-time monitoring of gait and balance, offering a promising alternative to manual clinical assessments. Similä et al. [9] developed a system that integrates an IMU with machine learning algorithms to forecast fall risk while automatically assessing BBS scores.

Despite the progress, current research still faces several challenges. Many existing methods rely on handcrafted features or simplified models that do not fully capture the complex dynamics of human gait, especially in populations with neurological impairments. Badura et al. [10] proposed a system with five IMUs and a machine-learning algorithm. The system of Shahzad et al. [11] only requires one IMU but the lasso regression, the method of the system, needs feature engineering. Lin et al. [12] used two IMUs and obtained a relatively good performance, but they also used a machine learning algorithm that required multiple experiments and manual comparisons to select the appropriate gait parameters. Deep learning methods that do not require manual feature extraction are in high demand. Additionally, the extraction of both spatial and temporal features remains areas that require further exploration [13] to achieve more accurate and robust balance evaluation.

In this paper, we proposed an automated BBS assessment system based on the deep learning method, which used IMU sensor data to compute BBS scores automatically. The proposed novel deep learning framework integrated convolutional neural networks (CNNs) for local feature extraction, bidirectional long short-term memory (Bi-LSTM) networks for capturing long-range temporal dependencies, and an attention mechanism to dynamically emphasize the most informative features. To validate the clinical utility of our system, we conducted comprehensive evaluations across diverse populations, including healthy individuals and patients with neurological diseases. This work laid the foundation for a new application of clinical assistance, transforming subjective scales into precision tools for neurorehabilitation.

## 2. Materials and Methods

### 2.1. System Overview

**Figure 1:**
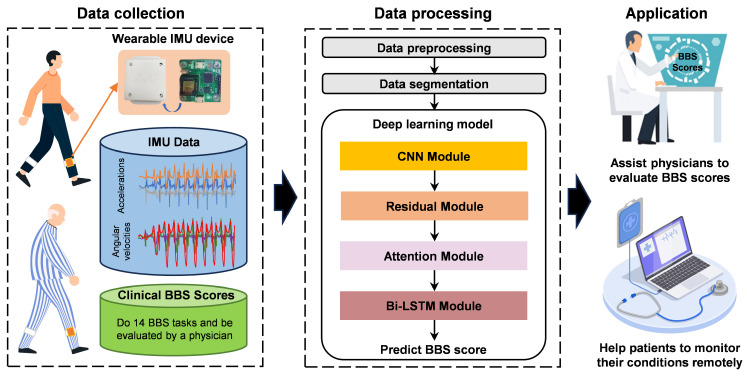
Schematic overview of the BBS assessment system. There are three main steps: data collection, data processing, and application. The orange line in the data collection shows the components of the wearable IMU device. The black lines in the data processing indicate which modules the data were processed through.

The proposed automated BBS assessment system integrated the wearable IMU device with a deep learning framework to objectively quantify balance performance. The overall work operated through three sequential phases as shown in Figure 1: data collection, data processing, and application.

Two types of data were collected during the data collection process. Firstly, the three-axis acceleration and three-axis angular velocity data collected by the IMU device on humans during walking were used to build the gait database. Then, the clinical BBS scores that were determined by a PT for each participant were recorded as the ground truth. During data processing, the gait dataset and scale scores were fed into the deep learning model for training. The final system can calculate the user’s BBS scores based on the walking data and be applied to assist healthcare physicians as well as patients in their own health monitoring [14].

### 2.2. Data Collection

#### 2.2.1. Information of Participants

We conducted the experiment to collect and process the data to create a gait and BBS score dataset. A total of 40 participants were recruited for data collection, 12 of which were young healthy control (HC) under 65 years old, 8 elderly HC over 65 years old, 8 patients with PD, and 12 stroke patients. The detailed characteristic information was shown in Table 1. For PD patients, most of them were in Stage I–II, indicating early disease with unilateral/bilateral involvement. UPDRS Part III scores < 32 typically reflected mild motor disability. All PD patients were evaluated using the MDS-UPDRS Part III during their “ON” medication state. For stroke patients, Modified Rankin Scale scores ≤ 2 indicated mild disability with preserved independence. All enrolled patients demonstrated only mild impairment levels, enabling full compliance with PTs’ instructions during the experiment. A physician supervised all data collection processes during the experiment to prevent accidents. All participants were informed and signed a consent form.

#### 2.2.2. Hardware Components of the IMU Device

**Figure 2:**
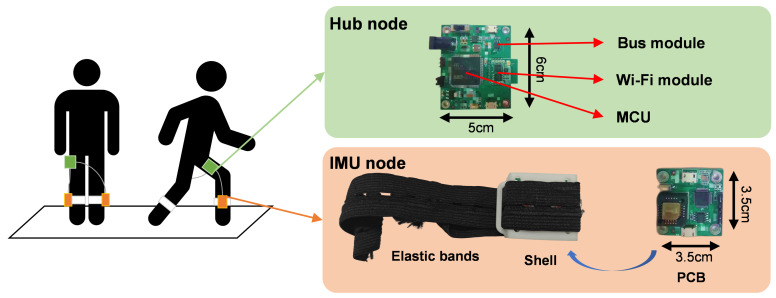
The locations and components of IMU device used in the system.

The hardware of this system included IMU devices. In order to minimize the impact of muscle movements on data collection during walking, the wearable IMU device was equipped with a hard shell created by 3-D printing. The outer side of the shell was secured to the human body by elastic bands to prevent sliding. The inner side of the shell contained the IMU node, which consisted of self-developed printed circuit board (PCB) [15]. This device also included a hub node to receive and transmit data from the IMU nodes to the host computer. Nodes were connected to each other by data cable. Figure 2 is a schematic diagram of the composition of a wearable IMU device, two IMU nodes were worn on the right and left calf of a person, and the hub node location was not required and was usually placed in the pocket.

The IMU module chip (YESENSE Technologies Co., Ltd., Wuhan, China) in the IMU node can measure 3-axis acceleration, 3-axis angular velocity, and 3-axis magnetic field data. In this study, we used 3-axis acceleration and 3-axis angular velocity data, so 3-axis magnetic field data was not sent to the host computer. The hub node consisted of three parts: a bus module, a Wi-Fi module, and a micro control unit (MCU), which transmited the IMU node signals wirelessly to the host computer via Wi-Fi. The sampling frequency was set to 100 Hz. The host computer received and stored the data using a self-developed C++20 program combined with a serial port debugging assistant.

#### 2.2.3. Task Protocol

**Figure 3:**
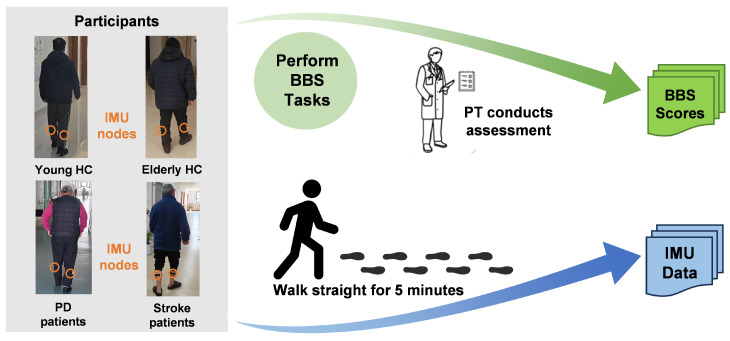
The workflow diagram of data collection, including clinical BBS scores collection and IMU data collection.

The data included ground truth BBS scores as well as walking IMU data. The workflow diagram of data collection was shown in Figure 3. For the BBS scores, a professional PT was present during the experiment to assess the participants’ clinical BBS scores. Each participant was required to complete 14 experimental tasks from the BBS, after which the PT would rate the completion of each task from 0 to 4, and the final total score would be the subject’s BBS score. The specific descriptions of the 14 tasks were described in the Table 2. For the walking IMU data, each participant was asked to walk for 5 min on a flat floor at a normal speed while wearing the device on the right and left calves. The IMU device would record the data during the walking process and transmit it to the computer.

### 2.3. Data Preprocessing

For the training of the deep learning model, the preprocessed IMU data would be used as input of the algorithm, and the BBS scores would be used as the ground truth.

#### 2.3.1. IMU Data Filtering and Standardization

The purpose of this step was to remove sensor noise and unify the data scale to improve model robustness. A low-pass Butterworth filter (4th order, cut-off frequency 20 Hz) was used to remove high-frequency noise [16]. Meanwhile, standardization was performed based on a static baseline (subjects were asked to stand still for 10 s to collect static data).

(1) $$X^{\prime }=\frac{X-\mu_{static}}{\sigma_{static}}$$

where *X* is the raw IMU data, $\sigma_{static}$ is the average of static data, $\sigma_{static}$ is the standard deviation of static data, and $X^{\prime }$ is the processed IMU data after standardization.

#### 2.3.2. BBS Normalization

Participants’ different balance functions may result in a wide range of scores, and in order to prevent the deep learning model from being affected by variations in the range of scores, this study used min–max normalization [17] to normalize the scores of the scale for training. The formula is as follows:

(2) $$S^{\prime }=\frac{S-S_{min}}{S_{max}-S_{min}}$$

where *S* is the unprocessed BBS score, $S_{min}$ is the minimum of the scale, in this case is 0, $S_{max}$ is the maximum scale of 56, and $S^{\prime }$ is the normalized BBS score.

#### 2.3.3. Gait Data Segmentation

**Figure 4:**
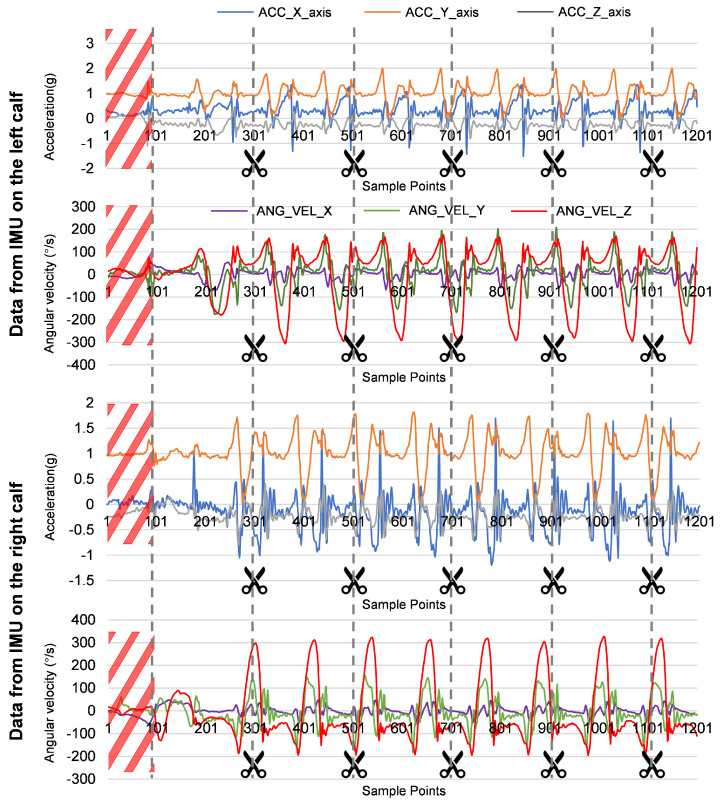
Example of the process of gait data segmentation. Inside the red slash box is the data to be deleted. The black dotted line means segment data in a fixed window size of 2 s.

During the data collection process, the first few seconds at the beginning of the collection and the last few seconds at the end of the collection had a higher rocking amplitude and a large difference in the data, so we truncated the data at both ends and use the first peak of the Z-axis angular velocity as the trigger point [18]. Then, in order to slice the continuous IMU signal into analyzable gait cycles and extract timing features, we segmented the data using sliding window segmentation. This method saved data processing time and increased the sample numbers for training. The length of a single gait cycle is about 1.6 s [19]. To ensure that a single window can contain all gait features, a fixed window size of 2 s was selected for segmentation to create the gait dataset. The total process was shown in Figure 4. Each participant had two IMUs on the body, and one IMU generated data for 6 axes with a sampling rate of 100 Hz, so the size of a segment data should be 200×12.

All data was preprocessed to generate a gait dataset of the same length to be fed into the deep learning model for training.

### 2.4. Deep Learning Model Architecture

**Figure 5:**
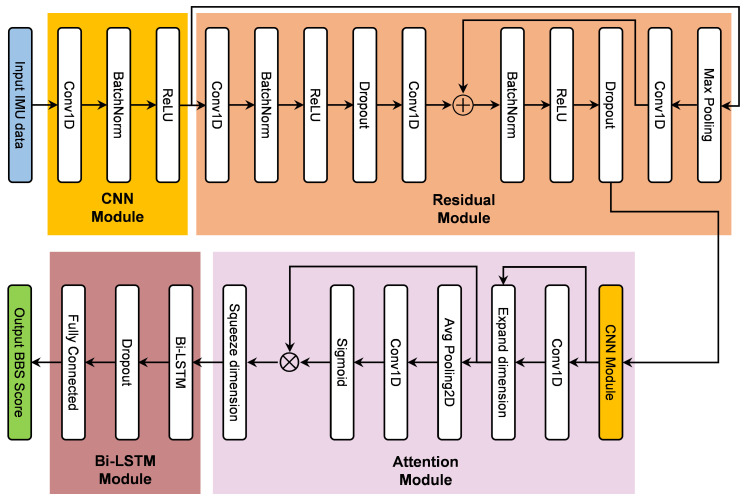
The architecture of the proposed deep learning model, mainly consists of the CNN module, the Residual module, the Attention module, and the Bi-LSTM module.

In this study, a model combining CNN and Bi-LSTM based on the Attention module was used to train and analyze the gait dataset and finally calculated the BBS score. The details of this model were shown in the Figure 5. It mainly consisted of four modules: CNN module, Residual module, Attention module, and Bi-LSTM module. The IMU data was processed sequentially through the four modules to output the predicted BBS scores.

Firstly, the CNN module was used for local feature extraction [20]. The processed segmentation of gait data was fed into the convolutional module, which included Conv1D, batch normalization, and the Rectified Linear Units (ReLU) activation function. Inertial data was processed using a one-dimensional convolution operation to construct a feature map. This one-dimensional convolution operation used kernel size 3 and filters 32.

Next the data was passed into the Residual module, which is used to deepen the features without losing gradient flow [21]. The data is processed through two routes and merged at the end of module. One processing route is to learn expanded features using a similar structure to the CNN module (one-dimensional convolutional layers, batch normalization, ReLU), and the other route uses max pooling to keep the input feature space. In the Residual module, there were convolution operations and would concatenate together. The kernel size was 3 and the filters size was 32. The dropout rate was set to 0.2.

Subsequently, the attention mechanism accentuated the most significant features by assigning appropriate weights to both the original and the expanded feature sets. Initially, this module increased the dimensionality of the input data to incorporate a richer contextual understanding before feeding both the raw and the enhanced data into the Efficient Channel Attention (ECA) block [22]. The maximum expanded dimension threshold value for dimension changes was set to 128. Function $F_{1}$ delineated a convolutional module that integrates convolution, batch normalization, and ReLU activation. Here, the operation denoted by *X* indicates that $y_{1}$ was constructed by combining the output from $F_{1}$ with the original input *X*. Moreover, function $F_{2}$ employed the ECA module to boost performance by accounting for each channel, along with its neighboring channels. The resultant data was then processed through a two-dimensional average pooling layer, followed by a one-dimensional convolutional layer and a sigmoid activation. Finally, function $F_{3}$ efficiently extracted the consolidated initial features while discarding the processed aggregated convolutional information, and the “squeeze” operation was used to reduce the dimensionality. The “squeeze” operation outputed a scalar with a dimension of 32.

(3) $$y_{1}=F_{1}\left(x,W_{1}\right)⊕x$$

(4) $$y_{2}=F_{2}\left(\sigma \left(CNN\left(y_{1}\right)\right)\right),k=\left|\frac{log_{2}\left(D\right)}{\tau }+\frac{a}{\tau }\right|$$

(5) $$y_{3}=squeeze\left(F_{3}\left(y_{2},W_{2}\right)\right)$$

where *x* means the input, $y_{1},y_{2},y_{3}$ are outputs at each stage, $F_{1},F_{2},F_{3}$ mean the operations in the module, $W_{1},W_{2}$ are weighting parameters of $F_{1},F_{3}$, σ is the Sigmoid function, kernel size *k* will be calculated by the channel dimension *D* and the hyperparameters τ and *a*.

The following Bi-LSTM module was used for capturing bidirectional temporal dependencies. The Bi-LSTM structure was proposed to fuse information from the past as well as messages from the future by using forward LSTM and backward LSTM [23]. The number of hidden units of forward LSTM and backward LSTM was set to 32. It took features from the attention module and processed them to produce the hidden states. After that came a dropout layer with the rate of 0.3 to discard some parameters and avoid overfitting. Finally, fully connected layers are used to generate the BBS scores.

## 3. Experiments and Results

### 3.1. Training Details

After processing the data and training the model, the performance of the model depended on the bias between the score of the scale of the patients in the clinical trial and the score of the scale predicted by the model. We adopted two evaluation metrics to assess the deep learning model’s performance: mean absolute error (MAE) and root mean square error (RMSE).

Mean absolute error:

(6) $$MAE=\frac{1}{n}\sum_{i=1}^{n}\left|{\hat{\beta }}_{i}-\beta_{i}\right|$$

Root mean square error:

(7) $$RMSE=\sqrt{\frac{1}{n}\sum_{i=1}^{n}{\left({\hat{\beta }}_{i}-\beta_{i}\right)}^{2}}$$

where $\beta_{i}$ means the actual score assessed by PT, ${\hat{\beta }}_{i}$ means the predicted score predicted by our model, *n* is the number of samples.

The dataset contained 4800 samples. Five-fold cross-validation was performed in training to ensure the reliability of the assessment and reduce the risk of overfitting, while the performance of each model was the average of the scores from the five experimental assessments. It divided the dataset into five similarly sized subsets (called “folds”, here it included 960 samples), and evaluated the model performance by rotating four of the folds as the training set and one of the folds as the test set, and finally combining the results of the five times. In total, 80% of the data (3840 samples) was used for training in each iteration, and 20% (960 samples) acted as the validation set in each iteration.

### 3.2. Results

**Figure 6:**
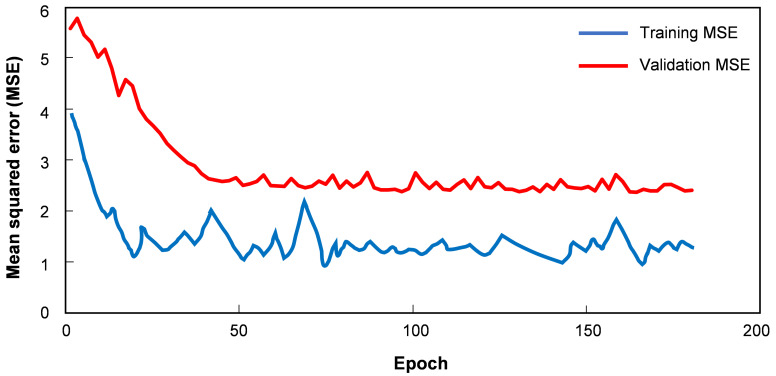
The learning curve of the training and validation loss (MSE).

The Adam optimizer was utilized with a learning rate of 0.001, and the mean squared error served as the loss function. Ultimately, a batch size of 64 and 200 epochs were chosen for training the deep learning model. Figure 6 showed the learning curve of the training and validation loss (MSE). Early stopping method is implemented, we set stopping halts training when validation loss stopped improving for 20 epochs. The training stopped at epoch 187 due to no improvement after a patience of 20 epochs, the optimal epoch would be 167.

To assess the impact of various modules on the model’s performance, we trained five models to compare the performance of the models with different combinations of modules. As shown in Table 3, the combination of CNN and Bi-LSTM makes RMSE from more than 2 to 1.8866, and MAE from more than 1.5898 to 1.5606. The use of the attention module also greatly improved performance. After the experiments, we got the lowest RMSE (1.5333) and MAE (1.1627) in the model using CNN and Bi-LSTM with attention.

**Figure 7:**
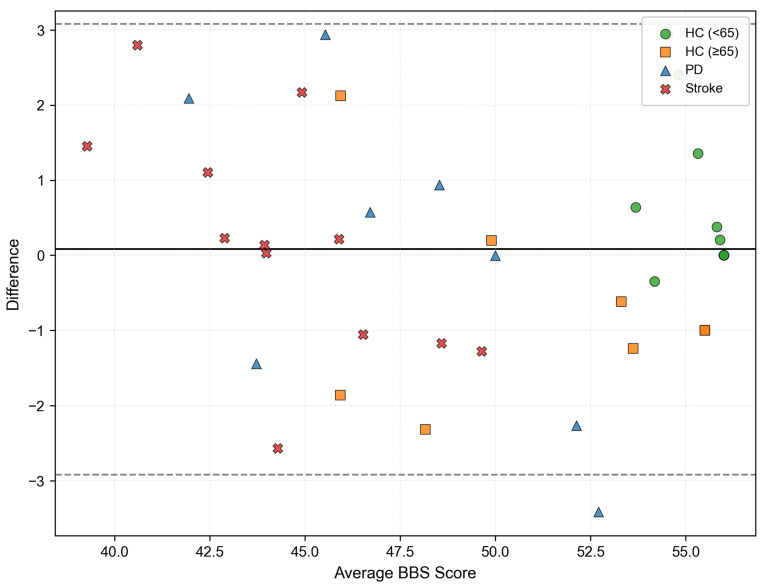
A Bland–Altman plot comparing the ground truth BBS scores with the BBS scores estimated by the proposed model. The solid black line indicates mean difference, and gray dotted lines represent 95% limits of agreement.

A Bland–Altman plot, shown in Figure 7, was used to compare our system’s predicted BBS scores with actual clinical BBS scores, showing an extremely high level of agreement between the two sets of data. A Bland–Altman plot is a valuable tool for checking if the measurement methods are sufficiently consistent for practical use. The x-axis represented the average of two groups of data. The y-axis represented the difference between the two groups. The solid black line was a mean difference line, which represented the average of all differences, and the two dotted lines meant limits of agreement (it is calculated by difference ± 1.96 times the standard deviation of the differences).

A near-total 97.5% (39/40) of all comparisons were within limits, indicating that the predicted result matches the PT’s scores and had a relatively high application value. Only one patient with PD had data that did not conform to the overall pattern, within an acceptable margin of error.

## 4. Discussion

We proposed a system for predicting BBS scores using a wearable IMU device and a deep learning algorithm, where the users simply walked for 5 min and their BBS score were determined using IMU data from the left and right calves. As shown in Table 4, the results of four different categories of people were verified in the experiment: young healthy people under 65 (RMSE: 1.1892, MAE: 0.6897), elderly healthy people above 65 (RMSE: 1.4702, MAE: 1.2943), patients with PD (RMSE: 2.0328, MAE: 1.7075), and patients with stroke (RMSE: 1.4923, MAE: 1.1847). Comparing the results of the algorithm across the different categories, the algorithm performed best in young healthy people and worst in patients with PD. Overall, the algorithm was more applicable to healthy people than patients with neurological diseases.

To demonstrate the contribution of this work, we compared the results obtained from this work with other relevant studies. While earlier studies laid important groundwork, our approach addressed critical gaps in sensor modality, user friendliness, participant diversity and performance. As shown in Table 5, prior systems predominantly relied on constrained sensing platforms: Johnson et al. [7] utilized Kinect2 cameras, limiting assessments to laboratory environments, while Shahzad et al. [11] and studies [12,24] adopted IMUs but focused on narrow task protocols (e.g., timed up-and-go or specific BBS sub-tasks). In contrast, our IMU-based framework analyzed natural walking patterns. By requiring only 5 min of walking data, our system avoided the artificiality of task-specific evaluations (e.g., ‘Task 5 and 6’ in [7]), aligning with the preference of clinicians for holistic assessments. Moreover, existing studies prioritized homogeneous cohorts: Johnson et al. [7], the studies [12,24] only tested HC, and Shahzad et al. [11] included only 23 participants (mixed HC and patients). Our work advanced generalizability by evaluating 40 participants, including healthy young/elderly adults and patients with PD or stroke. This diversity ensures robustness to pathological gait patterns absent in prior datasets. Notably, our MAE of 1.1627 and RMSE of 1.5333 outperformed other studies, including Shahzad et al.’s lasso regression (MAE = 1.44) [11], despite their narrower participant pools. In total, this work had the advantages of user friendliness, wide application scenarios, and good performance (low MAE and RMSE).

As for practical applications, from practitioners’ perspective, this system addresses pressing clinical needs through three advantages: time efficiency, objective monitoring, and telemedicine integration. Reducing the assessment time from 20–30 min to 5 min of walking data collection enabled PTs to allocate more time to personalized interventions while maintaining remote monitoring capabilities through online analytics. The continuous home-based evaluation paradigm would facilitate early intervention through two key mechanisms: (1) tracking of recovery trajectories (e.g., monitoring weekly BBS trends), and (2) fall prevention by detecting subtle balance deterioration.

For users, the system offered accessibility and transparency. The simplified protocol not only reduces clinic visit frequency—particularly valuable for telemedicine integration—but also minimizes learning barriers through natural walking tasks instead of complex BBS tasks. Visualized BBS trends serve dual purposes: empowering patients to understand their recovery progress while providing actionable insights for fall prevention through intuitive risk level indicators. This design aligns with telerehabilitation principles by enabling remote data interpretation that bridges clinic expertise with home-based care.

Nonetheless, the study had certain limitations. First, although the study recruited both healthy individuals and patients, all participants were capable of walking independently. Their balance proficiency surpassed that of individuals who rely on assistive devices for stability, suggesting that the model might not be applicable to those who require such equipment. Second, the study was limited to predicting aggregate scores on the scale rather than providing scores for each of the 14 individual tasks. Future research should focus on resolving these limitations to improve the system’s applicability in clinical settings.

## 5. Conclusions

In this work, a novel deep learning-based system designed to estimate Berg Balance Scale scores using IMU sensor data collected while walking was introduced. By integrating convolutional layers, Bi-LSTM networks, and an attention mechanism, our approach successfully captured both the local and long-term temporal features inherent in gait patterns. Experimental findings confirmed that the model accurately predicted BBS scores for both healthy individuals and patients with neurological diseases by recruiting 20 healthy subjects (12 young healthy subjects under 65 and 8 elderly healthy subjects over 65) and 20 patients (8 patients with PD and 12 patients with stroke). In comparison with other studies, it achieved lower MAE (1.1627) and RMSE (1.5333), suggesting its viability as a tool for balance assessment.

According to the results, this work can be applied to assist healthcare physicians as well as patients in their own health monitoring. This proposed system required only 5 min of walking data to perform assessment, while traditional assessment required participants do multiple tasks as well as the presence of the PT. It represents a significant step towards advancing assistive technologies for clinical care and establishes a blueprint for integrating wearable sensor data into digital management systems.

## Figures and Tables

**Table 1:** Characterization data of all participants.

	HC (≤65) (n = 12)	HC (≥65) (n = 8)	PD (n = 8)	Stroke (n = 12)
Age (years)	38.74 ± 12.43	72.38 ± 4.57	56.85 ± 8.41	60.73 ± 5.66
Height (m)	1.70 ± 0.18	1.66 ± 0.31	1.58 ± 0.26	1.63 ± 0.04
Weight (kg)	70.31 ± 8.24	60.68 ± 2.32	49.31 ± 4.83	55.71 ± 7.94
H&Y Staging	-	-	1.50 ± 0.76	-
UPDRS Part III	-	-	20.8 ± 5.6	-
Modified Rankin Scale	-	-	-	1.67 ± 0.89
BBS Scores	55.67 ± 0.75	50.63 ± 3.67	47.63 ± 3.04	44.50 ± 2.47

n means the number of different groups. The format of data is mean ± standard deviation.

**Table 2:** BBS test tasks.

Task Index	Description
1	Move from sitting to standing
2	Stand up unsupported
3	Sit unsupported
4	Move from a standing to a sitting position
5	Transfer from one chair to another
6	Stand up with eyes closed
7	Stand with two feet together
8	Reach forward with an outstretched arm
9	Pick an object up off the floor
10	Turn and look behind
11	Turn around in a complete circle
12	Alternate placing each foot onto the stool
13	Stand unsupported with one foot in front
14	Stand on one leg for as long as one can

**Table 3:** Performance comparison of different models.

	RMSE	MAE
CNN	2.0561	1.5898
Bi-LSTM	2.5789	2.0345
CNN + Bi-LSTM	1.8866	1.5606
Bi-LSTM (with attention)	1.8101	1.3819
CNN + Bi-LSTM (with attention)	**1.5333**	**1.1627**

Bold values denote the best results (lowest RMSE/MAE).

**Table 4:** Performance metrics stratified by participant subgroups.

Subgroup	RMSE	MAE
Young healthy people	1.1892	0.6897
Elderly healthy people	1.4702	1.2943
Patients with PD	2.0328	1.7075
Patients with stroke	1.4923	1.1847

**Table 5:** Comparison of the results of the algorithm proposed in this paper with other studies.

	Johnson et al. [7]	Shahzad et al. [11]	Lin et al. [12]	Lin et al. [24]	This Work
Device	Kinect2	IMU	IMU	IMU	IMU
Participants	43	23	136	136	40
Participant category	HC	HC and Patients	HC	HC	HC and Patients
Test task	Task 5 and task 6 in BBS	TUGT, FTSS and AST tasks ^1^	Task 12 and task 14 in BBS	Walking 15m	Walking
Model	Neural network	Lasso regression	Random forest	CNN + LSTM	CNN + Bi-LSTM (with attention)
BBS RMSE	N/A	1.9700	N/A	N/A	**1.5333**
BBS MAE	1.1677	1.4400	1.2700	1.4300	**1.1627**

^1^ TUGT is timed-up and go test, FTSS is five times sit-to-stand test and AST is alternate step test. Bold values denote the best results (lowest RMSE/MAE).

## Data Availability

The data relates to potentially identifiable patient information and sharing it would be a breach of the confidentiality agreement under which it was collected. The authors do not have permission to share the self-collected dataset.
